# PredatorHP Revamped (Not Only) for Interval-Sized Memory Regions and Memory Reallocation (Competition Contribution)

**DOI:** 10.1007/978-3-030-45237-7_30

**Published:** 2020-03-13

**Authors:** Petr Peringer, Veronika Šoková, Tomáš Vojnar

**Affiliations:** 8 Johannes Kepler University, Linz, Austria; 9 University of Birmingham, Birmingham, UK; Faculty of Information Technology, Centre of Excellence IT4Innovations, Brno University of Technology, Brno, Czech Republic

## Abstract

This paper concentrates on improvements of the PredatorHP shape analyzer in the past two years, including, e.g., improved handling of interval-sized memory regions or new support of memory reallocation. The paper characterizes PredatorHP’s participation in SV-COMP 2020, pointing out its strengths and weakness and the way they were influenced by the latest changes in the tool.

## 1 Verification Approach and Software Architecture

We first briefly recap the main ideas behind PredatorHP and then discuss significant improvements that have been done in the tool in the past two years.

### 1.1 The Predator Shape Analyzer

Predator is implemented using C++ and the Boost libraries as a GCC plug-in on top of the Code Listener framework [2], which we recently upgraded to work with GCC 7.4.0. Moreover, as shown below, we extended Code Listener by adding a type analysis phase before the compiled code is passed to the shape analysis implemented in Predator. In case a memory safety property is to be checked and there are no complex types, such as structures, unions, arrays, strings, or pointers in the program under analysis (including possibly unreachable code), we directly assume the program to be memory safe.

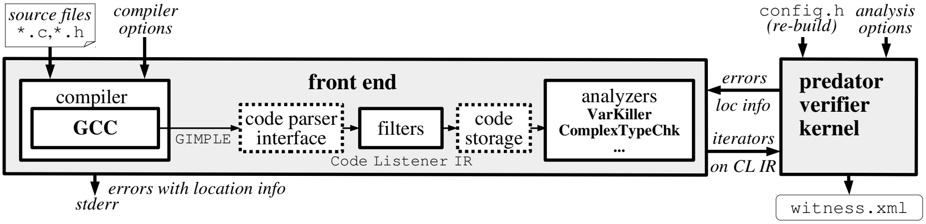

The main aim of Predator is *shape analysis* of sequential C programs that use low-level C pointer statements to implement various kinds of lists (singly- or doubly-linked, possibly circular, nested, and/or shared). Predator looks for various *memory-related errors* (invalid pointer dereferences, double free operations, memory leaks, etc.), and it

⋆ This work was supported by the Czech Ministry of Education, Youth and Sports within the IT4Innovations Excellence in Science (NPUII) project No. LQ1602.

⋆⋆ Jury member, email: `isokova@fit.vutbr.cz`.

A. Biere and D. Parker (Eds.): TACAS 2020, LNCS 12079, pp. 408–412, 2020.
https://doi.org/10.1007/978-3-030-45237-7_30

also checks validity of *assertions* present in the code. Predator uses *abstract interpretation* based on the domain of *symbolic memory graphs* (SMGs) [1]. Predator abstracts uninterrupted sequences of singly- or doubly-linked memory regions into appropriate kinds of list segments. Further, Predator abstracts numerical values (either values stored in memory regions, sizes of the regions, or offsets of pointers) using intervals with constant bounds. The constants used as the bounds have a pre-defined maximum/minimum value defined in the configuration of Predator (+32/-32 for SV-COMP'20). If the maximum/minimum value is exceeded, the bound is set to plus or minus infinity. Predator uses *summaries* to speed up analysis of programs structured into functions. Recursive programs are, however, analysed up to a given call depth only.

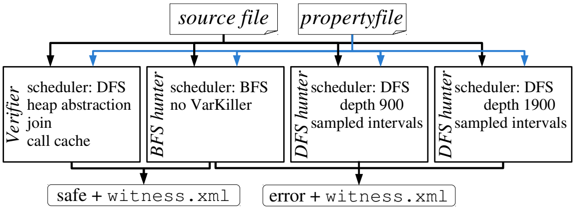

PredatorHP, i.e., the *Predator Hunting Party* [3,4], whose flow of control is shown on the right, is implemented as a Python script, and used to increase the efficiency and precision of the analysis. Namely, PredatorHP runs the base *Predator verifier* in parallel with several *Predator hunters* that do not use the list-segment abstraction, do not join semantically different SMGs, nor use function summaries with matching of call parameters based on SMG entailment. While the Predator verifier can claim a program correct, it cannot report errors to avoid false alarms caused by abstraction. Predator hunters are classified as *breadth-first* (BFS) and *depth-first* (DFS). The DFS hunters have a limit on the search depth defined as a certain number of GCC's GIMPLE instructions. The hunters can normally only report errors. The only exception is when the verified program has a finite state space that is fully explored by the BFS hunter in the given time limit.

In SV-COMP'20, based on empirical data, the BFS hunter does not use the Predator's *VarKiller*, which removes dead variables from SMGs. This led to a significant speedup on 5 verification tasks (and some slowdown on 3 tasks). Further, the most shallow DFS 200 hunter, searching up to the depth of 200 instructions and used in PredatorHP up to SV-COMP'19, was removed as it was not bringing any advantage wrt the DFS 900 hunter, and a DFS 1900 hunter was added to handle more complex tasks (in particular, `memsafety-ext2/split_list_test05-1`, `ntdrivers/floppy.i.cil-3`). However, note that the DFS 900 hunter remains needed as otherwise 11 verification tasks would time out.

## 1.2 Recent Modifications of PredatorHP

One of the main improvements of the latest version of Predator is that its SMG-based analysis has been extended to support *memory reallocation* on the heap. If a reallocation operation is executed on an SMG, two new SMGs are produced. The first one models the case when a new object of the required size is created, data from the old object are copied into the new object, and the old object is freed. In the second case, the existing object is resized. If the size decreases, Predator checks that no memory leak happens due to some pointer field is removed or invalidated (in case it is partially removed).

Another improvement concerns working with *interval-sized memory regions*, which arise when allocating structures or arrays of parametric size. Despite even older versions of Predator were able to create such regions, the way in which they could have been treated in the subsequent analysis of the program was very limited. In particular, it was impossible to dereference interval-sized regions, and hence Predator was very weak when analysing programs with structures or arrays of an in-advance-not-fixed size. This situation was first improved for SV-COMP'19 in the following pragmatic way.

Namely, whenever Predator hits a conditional statement that would previously yield an interval value with fixed bounds (such as the statement `if (n>=0 && n<10)` for so-far unconstrained `n`), it will split the further analysis into as many branches as the number of values in the interval is, each of them evaluating for a concrete value from the interval. After the split, no further interval-based allocations and dereferences, which the previous version of Predator used to fail on, happen. In order for the splitting not to cause a memory explosion, the latest version of Predator contains a parameter that controls the maximum size of split intervals, which was set to 300 in SV-COMP'20.

The above modification of Predator concerned dealing with memory regions whose size is given by an interval with finite bounds. In case one of the bounds is infinite, Predator has been extended to *sample* the interval and perform the further analysis with the sampled values. Currently, the sampling is done simply by taking some number of concrete values from the given interval starting/ending with the bound that is fixed (of course, for memory regions, unboundedness from above does only make sense). The number of considered samples is currently set to 3. Of course, this strategy cannot be used to soundly verify correctness of programs, and so it is used for detecting bugs only.

Despite the above mentioned treatment of intervals was primarily designed for dealing with interval-sized memory regions, it can help in other cases of dealing with integers too. Namely, it can help both when dealing with integer data as well as when dealing with interval-based pointer offsets.

Next, we have implemented checking whether all dynamically allocated memory has been deallocated when a function with the *noreturn attribute* (such as `abort` or `exit`) is called. The implementation simply searches the SMG representing the memory at the moment of a call of a noreturn function and checks that it does not contain any valid dynamically allocated object.

We have also added a support of the *clobber* instruction of GIMPLE, which terminates the life time of local variables of code blocks. Upon this instruction, Predator now marks the concerned memory region as deallocated, allowing it to detect *invalid dereferences* of *objects local to a block* from outside of the block. Further, we have added a support of the instructions *modulo* and *bitwise-or* and created models of the standard library functions for `strcmp` and `realloc`. This fixed several problems such as reporting false alarms when assigning fully-overlapping structures.

Finally, we improved the generation of witnesses. Apart from some bug fixes, we changed the trace generation for the reachability category. Namely, in this category, if some trace ends with an error other than calling `__VERIFIER_error`, the analysis recovers and continues to search for other traces.

## 2 Strengths and Weaknesses

The main strength of PredatorHP is that it treats code with various kinds of unbounded lists in a *sound* and *efficient* way. Predator hunters then allow it to quickly handle programs with a small finite state space (e.g., benchmarks from `list-simple`) and avoid many false alarms that could otherwise happen. Interestingly, among the 328 correct tasks in *ReachSafety-Heap*, *MemSafety-Heap*, and *MemSafety-LinkedLists*, only 98 use unbounded data structures, out of which the Predator verifier (and, of course, no hunter) handles 56 %. Next, out of the 328 tasks, 83 do not use linked data structures nor arrays, and 147 use them but are finite-state. The Predator verifier and the BFS hunter handle 93 % of the 83 tasks that are so trivial that even the verifier does not use any abstraction. Out of the 147 tasks, 53 tasks are handled by both of them, while 2 tasks are handled solely by the verifier and 75 solely by the BFS hunter.

A weakness of Predator is that it specialises in dealing with lists, and so it handles structures such as trees, skip-lists, or arrays in a bounded way, i.e., for error detection, only. Another weakness of Predator has traditionally been its weak treatment of non-pointer data. We have tried to improve on the latter weakness by the described heuristics for dealing with intervals of integers with a specific aim to improve the way Predator handles memory regions of parametric size. The results of PredatorHP on SV-COMP'20 benchmarks with arrays show that the heuristics did help. Indeed, the interval sampling heuristic allowed us to correctly detect 10 errors in tasks from `array-memsafety`, `array-examples`, and `loops`. Moreover, the interval-splitting heuristic also helped on some benchmarks for dealing with interval-based sizes, offsets, and/or integer data. Namely, it removed 8 unknown results in *ReachSafety* and 4 such results in *MemSafety*.

The new type analysis looking for presence of complex types allowed Predator to skip its main analysis loop in 77 tasks in the *MemSafety* category, of which 13 tasks (from `termination-crafted`) contain recursion, which Predator could not handle, and 6 tasks (from `locks`) would otherwise timeout. Due to the new support of reallocation, Predator verifies all tasks containing a call of `realloc`. Due to the added support of `clobber` instructions, Predator detects invalid memory accesses in benchmarks accessing variables outside of the block in which they were created. All other new improvements described above did also help in some cases and allowed PredatorHP to win the 1st place in the *MemSafety* category and in the *ReachSafety-Heap* sub-category.

## 3 Contributors, Software Project, and the Tool Setup

The main author of Predator is Kamil Dudka. Besides him and the PredatorHP team, Petr Müller, Michal Kotoun, and numerous other people listed in the `docs/THANKS` file in the distribution of Predator have contributed to the distribution of Predator.

Predator is an open source software project distributed under GNU GPLv3. The source code used in SV-COMP'20 is available too[1]. The `README-SVCOMP-2020` file shipped with it describes how to build the tool. The script `predatorHP.py` serves to run the tool, taking a verification task file as a single positional argument. Paths to both the property file and the desired witness file are accepted via long options, i.e., 64-bit compiler options. The verification outcome is printed to the standard output. To run PredatorHP in the BenchExec environment, the `predatorhp.py` wrapper and the `predatorhp.xml` benchmark definition can be used. In SV-COMP'20, PredatorHP participated in the *MemSafety* category and in the *ReachSafety-Heap* sub-category.

[1] http://www.fit.vutbr.cz/research/groups/verifit/tools/predator-hp
